# Salmon protein hydrolysates and upcycled oil as functional ingredients for hybrid oat okara spreads: a circular marine by-product valorization approach

**DOI:** 10.3389/fnut.2026.1914478

**Published:** 2026-08-17

**Authors:** Daniela Lorena Lamas, Ingrid Aguiló-Aguayo

**Affiliations:** 1 Institute of Marine and Coastal Research IIMyC-CONICET, Mar del Plata, Buenos Aires, Argentina; 2 IRTA, Postharvest, Fruitcentre, Lleida, Catalonia, Spain

**Keywords:** Atlantic salmon by-products, circular bioeconomy, food upcycling, hybrid emulsions, oat okara, protein hydrolysates, texture-modified functional foods

## Abstract

**Introduction:**

Upgrading fishery and agro-industrial by-products represents a critical frontier in circular food engineering and precision nutrition. This study aimed to design and characterize a novel, nutrient-rich, functional texture-modified semi-solid paste based on micro-milled oat okara as a structural biopolymer core and Atlantic salmon (Salmo salar) protein hydrolysates (SPH) obtained by cascade enzymatic hydrolysis as protein-rich ingredients with potential interfacial activity.

**Methods:**

Two multi-enzyme systems, System A (Alcalase^®^ 2.4 L + Flavourzyme^®^ 500 MG) and System B (Protana^®^ Prime + Flavourzyme^®^ 500 MG), were applied to freeze-dried salmon trimmings with a water ratio of 1:3 at 50 °. Chromatographic profiling (GC-FAME), instrumental texture profile analysis (TPA), and colorimetry were performed to evaluate lipid preservation and structural characteristics.

**Results:**

Extensive mass-balance monitoring revealed precise macroscopic nitrogen solubilization degrees (DH_N_) of 34.72 ± 1.28% for System A and 26.99 ± 1.12% for System B, with both operations simultaneously recovering over 70 mL of pure upcycled salmon oil. Chromatographic profiling (GC-FAME) confirmed that oxidation-prone marine omega-3 fatty acids, including eicosapentaenoic (EPA) and docosahexaenoic (DHA) acids remained entirely undamaged and preserved during processing. Integrating SPH into the oat okara network successfully enabled the production of stable hybrid spreads with a robust protein density ranging from 17% to 24% dry weight and a controlled emulsified fat base of around 6% wet basis. Instrumental texture profile analysis (TPA) and colorimetry showed that pure liquid peptides acted as structural plasticizers to enhance creaminess, dropping hardness down to 1.79 N. Conversely, the strategic reincorporation of 3% upcycled salmon oil functioned as an active filler, reinforcing the biopolymer network through interfacial anchorage onto insoluble oat fibers, maximizing hardness to 3.94 ± 0.96 N and increasing optical lightness (L^*^ = 64.05).

**Discussion:**

The smooth texture and outstanding physical stability near neutrality highlight the promising future potential of these hybrid matrices as functional texture-modified foods for specialized nutrition.

## 1 Introduction

Driven by the increasing demand for sustainable resource management and circular bioeconomy strategies, marine by-products have gained considerable attention as sources of high-value bioactive compounds. Globally, the commercial seafood processing sector generates an estimated 20 to 35 million tons of fish waste annually, which aligns with estimates that approximately 35% of total global catches are lost or discarded across the supply chain (1). This massive accumulation represents an immense economic and environmental challenge that urgently demands advanced bio-refinery interventions. Among them, Atlantic salmon (*Salmo salar*) represents one of the most important aquaculture species worldwide, generating substantial quantities of processing by-products (2). During industrial fileting and processing, it is estimated that 40% to 60% of the total salmon biomass including heads, frames, skin, viscera, and trimming cuts remains underutilized despite being exceptionally rich in proteins, bioactive peptides, essential amino acids, collagen, and omega-3 fatty acids (3, 4). The conversion of these by-products into functional food ingredients offers an attractive opportunity to increase resource efficiency and create value-added products for human consumption (3–6).

Enzymatic hydrolysis has emerged as one of the most effective approaches for upgrading fish-processing by-products. Protein hydrolysates obtained from salmon residues contain peptides with antioxidant, antihypertensive, anti-inflammatory, and techno-functional properties, including emulsifying and water-holding capacities, making them attractive ingredients for food formulation (4, 7, 8, 38).

In parallel, the expanding plant-based beverage industry generates substantial volumes of oat okara, a highly nutrient-dense yet drastically underutilized solid side-stream (9, 10). This cereal by-product has a unique macromolecular profile, being rich in insoluble dietary fibers and structural components. However, its application in complex food systems is fundamentally limited by several factors. The first problem that must be addressed for its subsequent inclusion in food products is the perishability and short shelf life of oat okara. Thus, high-pressure pasteurization reduces the microbiological content of this biomass, thereby improving its shelf life. However, this processing method affects the residue's overall composition, causing partial protein denaturation and consequently reducing viscosity while increasing soluble fiber content (10, 11). Raw oat okara matrices lack the cohesive rheological networks necessary to stabilize multi-phase systems, such as emulsified spreads. To overcome these functional bottlenecks and successfully upcycle this biomass beyond traditional low-value uses, recent literature highlights the necessity of implementing physical, chemical, or enzymatic modifications capable of releasing entrapped nutrients and modifying macrostructural behavior (10). In this context, the synergistic integration of pre-conditioned oat okara with small-peptide fractions from highly functional marine protein hydrolysates represents a novel circular food design strategy to repair the structural and mechanical gaps of the vegetable cell-wall network while significantly elevating its nutritional profile.

The combination of salmon protein hydrolysates and oat okara represents a promising strategy for developing hybrid foods that integrate marine and plant resources within a circular economy framework.

The development of highly hydrated, nutrient-dense hybrid matrices offers a compelling avenue to design advanced texture-modified foods. Populations requiring tailored rheological textures, such as individuals experiencing transient swallowing difficulties or elderly groups prone to muscle wasting, frequently suffer from severe nutritional dilution and low protein bioavailability in their diets (12). Meeting their requirements demands homogeneous, cohesive, and phase-stable semi-solid matrices that minimize the risk of bolus separation. In this context, the synergistic integration of a pre-conditioned oat okara structural base with small-peptide fractions from functional salmon protein hydrolysates represents an innovative food engineering strategy. The fish-derived peptides may contribute to interfacial stabilization at oil-in-water interfaces, potentially compensating for structural weaknesses within the pasteurized plant cell-wall matrix and reducing the risk of syneresis. While not clinically standardized under specific diagnostic frameworks, the resulting soft texture and balanced viscoelastic behavior suggest a promising future potential for these hybrid matrices in the field of design-driven, texture-modified functional nutrition (13).

Therefore, this study aimed to valorize Atlantic salmon (*Salmo salar*) processing by-products through the production of functional protein hydrolysates generated by sequential multi-enzyme systems. Alcalase^®^ 2.4 L was selected for its robust endopeptidase activity to maximize initial protein solubilization, and Protana^®^ Prime was chosen for its specific exopeptidase profile designed to release non-bitter terminal fractions. Both systems were combined with Flavourzyme^®^ 500 MG in a secondary stage to refine the peptide distribution, mitigate potential sensory bitterness and evaluate the technological application of the hydrolyzates as natural emulsifying ingredients in a hybrid oat okara-based spread. The central hypothesis guiding this research was that incorporating amphiphilic marine peptides alongside upcycled salmon oil would repair the structural gaps in the pasteurized plant cell wall matrix functionally, with the lipid droplets operating as active fillers to establish a cohesive, creamy and colloidally stable functional food structure that does not require synthetic texturizers. The physicochemical composition, antioxidant properties, and techno-functional performance of the hydrolyzates were characterized and subsequently assessed within semi-solid plant–marine formulations that differed in terms of their peptide profile and lipid composition (sunflower oil and salmon oil blends). The resulting hybrid spreads were evaluated in terms of their physicochemical characteristics, color, emulsion stability, and texture to determine the feasibility of converting marine and cereal by-products into functional foods.

## 2 Materials and methods

### 2.1 Raw materials

Atlantic salmon (*Salmo salar*) trimming by-products were sourced from an industrial fileting facility (Almacén de pescados JUÁREZ S. L. Burgos, Spain). The salmon trimmings were collected immediately after industrial fileting and transported to the laboratory within insulated thermal bags packed with crushed ice, strictly maintaining a temperature window of 0 to 4 °C to prevent native enzymatic autolysis and lipid oxidation. Upon arrival, samples were immediately stored at −20 °C prior to subsequent freeze-drying operations. The pasteurized agro-industrial oat okara biomass was supplied by Fruselva Global S.A.U provided it. (Reus, Catalonia, Spain) and transported directly to the laboratory utilizing a dedicated cold-chain courier service strictly maintained at 4 °C to avoid microbial proliferation or native carbohydrate fermentation before subsequent pre-conditioning operations The material was stored frozen at −20 °C immediately. Before formulation and analysis, samples were thawed at 4 °C for 16 h and mechanically homogenized to ensure phase and matrix uniformity. The moisture content was rigorously monitored to ensure formulation and legal consistency.

The enzymes Alcalase^®^ 2.4 L, Protana^®^ Prime, and Flavourzyme^®^ 500 MG were provided by Novozymes. Commercial corn oil (Koipesol, Spain) and food-grade ingredients, including sodium chloride and L-ascorbic acid from PanReac AppliChem (Barcelona, Spain), were used. Refined garlic powder (Ducros, Spain) and xanthan gum (Sigma-Aldrich, St. Louis, MO, USA) were incorporated as functional ingredients for the formulation.

### 2.2 Salmon by-products and elemental analysis

The proximate composition of the raw salmon by-products was determined in accordance with official standard protocols (14). Moisture Content was quantified gravimetrically via thermal loss in a convective oven at 105 °C until a constant mass was reached. Crude Lipids were extracted via the Bligh and Dyer (15) method. Ash Fraction was determined by complete calcination of the organic matter in a muffle furnace at 550 °C. Crude Protein: Total Nitrogen (N) content was determined via the Dumas combustion method. The total protein content was mathematically derived from the nitrogen fraction using the specific conversion factor (N × 6.25). To evaluate the comprehensive chemical and structural profiles, the raw elemental composition, specifically Total Carbon (C), Hydrogen (H), Nitrogen (N), and Sulfur (S) of the salmon byproducts was determined by elemental microanalysis in an EA Flash 2000 apparatus (Thermo Scientific: Waltham, MA, United States), equipped with a thermal conductivity detector (TCD). Oven temperature was set at 900 °C and gas flows were 250 mL/min for oxygen and 140 mL/min for helium, used as the carrier gas. A second flow of helium (100 mL/min) was used as a reference for the detector, whereas the calibration curve was prepared with different concentrations of 4-Aminobenzenesulfonamide. The amino acid profile was analyzed using gas chromatography (Hewlett-Packard 6890 series) with flame ionization detection (GC-FID). Prior to analysis, derivatization of the amino acids was required to increase their volatility, enabling them to be analyzed by GC without the need for excessively high temperatures, which would otherwise lead to thermal degradation. Derivatization involves a mixture of ethanol and pyridine, norvaline as the internal standard, propyl chloroformate (PCF) as the derivatization agent, and chloroform containing 1% PCF as the organic phase. An aliquot of 4 μL of the organic phase were injected at a split ratio of 1:15 at 250 °C into a ZB-AAA column (10 m × 0.25 mm I.D., Phenomenex Inc.: Torrance, CA, United States). The initial temperature of 110 °C was maintained for 1 min, after which the oven temperature was programmed to increase from 110 to 320 °C at a rate of 32 °C/min. Helium was used as the carrier gas at 60 kPa, and nitrogen as the make-up gas. The detector temperature was set at 320 °C. The amino acid profile was determined after hydrolysis using 6 N HCl at 100 °C for 24 h, followed by further derivatization. Since tryptophan and cysteine are lost by acid hydrolysis and methionine can be partially destroyed, alkaline hydrolysis using 4.2 N NaOH was also carried out to determine these amino acids.

### 2.3 Characterization of oat okara

To characterize the oat okara biomass, crude lipids were first extracted via the Soxhlet method using hexane as the solvent (Buchi B-811). The compositional profile of the oat okara was then comprehensively mapped in accordance with the standard Laboratory Analytical Procedures (LAPs) developed by the National Renewable Energy Laboratory (NREL). The moisture and ash content was quantified via gravimetric thermal drying at 105 ± 2 °C until a constant mass was reached (NREL/TP-510-42621). The structural inorganic ash fraction was determined by complete calcination in a muffle furnace at 575 ± 25 °C (NREL/TP-510-42622). To obtain the extractives and structural components, the soluble non-structural materials (including the water- and ethanol-soluble extractive fractions) were separated from the raw biomass and isolated using a continuous Soxhlet extraction apparatus over a 24-h cycle (NREL/TP-510-42619). Extractives-free okara samples were subjected to a two-stage from concentrated to dilute sulfuric acid (H_2_SO4) hydrolysis protocol to analyze acid hydrolysis of lignin and carbohydrates: Stage 1: 72% w/w at 30 °C for 60 min; Stage 2: dilution to 4% w/w, followed by autoclaving at 121 °C for 60 min (NREL/TP-510-42618). Acid-insoluble lignin (Klason lignin) was recovered from the hydrolyzed residue and determined gravimetrically. Conversely, the acid-soluble lignin remaining in the liquor was quantified via ultraviolet–visible (UV–Vis) spectrophotometry at a wavelength of 240 nm. The monomeric structural sugars (glucose, xylose, and arabinose) released into the hydrolysis liquor were identified and quantified using a high-performance liquid chromatography (HPLC) system equipped with a refractive index detector (RID) and a carbohydrate-specific separation column operating with high-purity standards.

To evaluate the comprehensive chemical and structural profiles, the raw elemental composition, specifically Total Carbon (C), Hydrogen (H), Nitrogen (N), and Sulfur (S) was determined via high-temperature combustion using a calibrated organic CHNS elemental analyzer using exactly 1.0 ± 0.1 mg of sample. Amino acids profile was also determined as described above.

### 2.4 Production and characterization of salmon protein hydrolysates (SPH)

The enzymatic conversion of the raw Atlantic salmon (*Salmo salar*) trimmings was executed utilizing two distinct multi-enzyme cascading configurations to evaluate comparative functional performances: System A (Alcalase^®^ 2.4 L followed by Flavourzyme^®^ 500 MG; AL + FL) and System B (Protana^®^ Prime followed by Flavourzyme^®^ 500 MG; PP + FL).

The primary endopeptidase treatment for System A relied on Alcalase^®^ 2.4 L, a broad-specificity serine endopeptidase derived from *Bacillus licheniformis* widely recognized for its capability to efficiently cleave interior peptide bonds, particularly at hydrophobic residues, thereby enhancing initial protein solubility and compressibility (16). Conversely, System B utilized Protana^®^ Prime, a proprietary commercial blend optimized for specific exopeptidase actions that combines targeted aminopeptidase and carboxypeptidase activities to release free amino acids from peptide terminal ends, improving overall nutritional bioavailability (16, 17). Both setups concluded with a common secondary hydrolysis stage utilizing Flavourzyme^®^ 500 MG produced by *Aspergillus oryzae*. This industrial enzyme complex possesses synchronized endo- and exopeptidase mechanisms, notably led by specific aminopeptidases and carboxypeptidases, allowing for internal peptide cleavage alongside the systematic removal of terminal residues to successfully enhance final product flavor profiles and mitigate potential sensory bitterness (16, 18).

For the hydrolysis procedure, the raw materials were thawed and approximately 250 g of Atlantic salmon trimming by-products were mixed with distilled water at a ratio of 1:3 (w/v) and homogenized at 50 °C. For all experimental runs, the multi-enzyme dosages were fixed at an enzyme/substrate ratio of 1% (w/w). This concentration was selected to establish a robust and commercially viable baseline, as previously validated in recent fish by-product valorization literature (17, 19). Furthermore, this specific 1% dosage aligns with the manufacturer's (Novozymes: Bagsvard, Denmark) recommendations for achieving optimal catalytic efficiency without triggering hyper-proteolysis, which could lead to excessive peptide degradation, severe bitterness, and sensory impairment of the final food matrix.

System A: The raw salmon slurry was initially adjusted to pH 8.0 and heated to 50 °C. Alcalase^®^ 2.4 L was added at 1% enzyme/substrate ratio and allowed to react for 60 min. Subsequently, the medium was adjusted to pH 7.0 at 50 °C, and Flavourzyme^®^ 500 MG at 1% enzyme/substrate ratio was introduced for a second hydrolysis stage of 60 min to promote debittering and flavor development.

System B: The salmon matrix was adjusted to pH 6.5–7.0 and heated to 55 °C for the initial extraction phase using Protana^®^ Prime at 1% enzyme/substrate ratio for 60 min. Following this, the parameters were set to pH 7.0 and 50 °C to initiate the sequential hydrolysis phase with Flavourzyme^®^ 500 MG at 1% enzyme/substrate ratio for 60 min.

Upon completion of the required reaction periods, the targeted proteases were thermally inactivated by subjecting the reactor to a heat treatment at 85 °C for 15 min. The crude slurry was subsequently centrifuged at 4,000 rpm for 20 min to isolate and collect the upper oil phase, SO the purified liquid SPH and the residue.

#### 2.4.1 Recovery yield of hydrolysis products

Following enzymatic hydrolysis and centrifugation, the bioprocess systematically generated three distinct phases: an upper salmon oil layer, a continuous liquid protein hydrolysate supernatant, and a heavy residual solid pellet. The mass-balance extraction yield of each recovered stream was mathematically calculated relative to the initial dry biomass weight of the raw salmon trimmings subjected to hydrolysis according to Equation 1:


$$\begin{array}{l} \text{Yield}\left(\%\right)=\text{mf}/\text{mi}\times 100 \end{array}$$ (1)

where mf represents the definitive mass of the targeted recovered fraction (oil, liquid hydrolysate, or solid residue) and mi is the baseline initial dry mass of the raw salmon substrate entering the bioreactor.

Specifically, the recovery performance of the upcycled marine oil was derived from the absolute mass of the isolated lipid phase separated after centrifugation and expressed as a cumulative percentage of the lipid content initial dry salmon input.

Concurrently, the total dissolved solubilized solids recovery within the continuous aqueous phase was determined gravimetrically by drying liquid aliquots at 105 °C to a constant weight, with solute recovery metrics normalized against the initial dry fish mass.

Finally, the net protein recovery yield within the continuous liquid supernatant was established based on the total nitrogen (TN) density tracked via high-temperature combustion analysis (TOC/TN analyzer) according to the ISO 20236 standard method (20). Taking into account the operational analytical dilution configurations, the total mass of elemental nitrogen extracted into the supernatant was converted to crude protein equivalents utilizing the standard marine conversion factor of 6.25. The definitive protein recovery yield was mathematically defined as the stoichiometric ratio of total solubilized protein mass in the liquid phase to the initial raw protein load entering the bioreactor, which was derived from the verified baseline crude protein concentration of the raw Atlantic salmon trimmings (43.11% d.b.).

### 2.5 Characterization of salmon protein hydrolysates

The liquid salmon protein hydrolysates were comprehensively characterized according to the following technological and chemical parameters:

Soluble Protein Quantification: Soluble proteins and low molecular weight peptides in the liquid SPH were quantified spectrophotometrically utilizing the Lowry (37) method (1951). An aliquot of 100 μL of the sample was use for each determination, and bovine serum albumin (BSA) was use as the standard calibration curve.

Total organic carbon and total nitrogen. The concentration of total organic carbon (TOC) and inorganic carbon (IC) was quantified using a Shimadzu TOC-V analyzer. Potassium hydroxide was used as a standard. Sodium phthalate and bicarbonate were also used as standards. The TOC concentration was then calculated by subtracting the IC concentration from the TC concentration obtained. The total nitrogen (TN) content was determined using KNO3 as a standard.

To evaluate the extension and catalytic efficiency of the sequential enzymatic treatments, the Degree of Hydrolysis (DH _N_ %) was quantified macroscopically based on the Nitrogen Solubility Index (NSI) protocol adapted for fish processing side-streams (21, 22). This foundational index defines the ratio of total nitrogen successfully liberated into the continuous liquid phase against the absolute elemental nitrogen load entering the bioreactor. The operational DH_N_ was mathematically resolved according to Equation 2:


$$\begin{array}{l} {\text{DH}}_{\text{N}}\left(\%\right)=\left(\left({\text{TN}}_{\text{liquid}}\times {\text{V}}_{\text{liquid}}\right)/\left({\text{N}}_{\text{raw}}\times {\text{m}}_{\text{raw}}\right)\right)\times 100 \end{array}$$ (2)

where TN_liquid_ corresponds to the total nitrogen concentration measured via high-temperature combustion analysis within the liquid phase, V_liquid_ is the net volume of liquid hydrolysate recovered after centrifugation (L); N_raw_ is the native elemental nitrogen percentage of the raw Atlantic salmon trimmings, determined via Dumas and m_raw_ is the initial dry mass of the marine by-product introduced into the bioreactor (g). Concurrently, to complement this macro-structural solubilization baseline, the spectrophotometric Lowry (37) assay was executed in parallel to quantify the specific concentration of soluble protein and peptide fragments within the continuous liquid phase.

Amino Acid Profile: The comprehensive amino acid composition was analyzed using gas chromatography (Hewlett-Packard 6,890 series) with flame ionization detection (GC-FID) as described previously in Section 2.2.

Antioxidant Capacity Assessment via FRAP Assay The selection of the Ferric Reducing Antioxidant Power (FRAP) assay as the primary antioxidant screening tool was methodologically driven by its specific capability to evaluate single-electron transfer (SET) mechanisms (reducing power) in highly soluble, low-molecular-weight peptide fractions (23). This colorimetric mechanism provides a direct, biochemically relevant indicator of a peptide pool's capacity to interrupt radical chain reactions and defensively delay the auto-oxidation of vulnerable polyunsaturated omega-3 fatty acids within multi-phase emulsified systems (24). The ferric reducing activity of the liquid salmon protein hydrolysates (SPH) was quantified by monitoring the structural reduction of a ferric tripyridyltriazine complex (Fe^3+^-TPTZ) to its intensely blue ferrous form (Fe^2+^-TPTZ) at 593 nm. An aliquot of 50 μL of the sample was use for each determination. The absolute antioxidant capacity was calculated and expressed as millimoles of ferrous sulfate equivalents per liter of liquid hydrolysate (mmol FeSO4/L) utilizing a rigorous external standard calibration curve according to Equation 3:


$$\begin{array}{l} {\text{Antioxidant Capacity mmol FeSO}}_{4}/\text{L}=\left(\Delta A_{593}-\text{b}/\text{m}\right)\times \text{DF} \end{array}$$ (3)

where Δ A_593_ is the net absorbance displacement of the sample (absorbance of the reaction mixture minus the reagent blank), m and b represent the analytical slope and y-intercept derived from the linear regression curve of the pure FeSO4·7H_2_O standards (0.1–2.0 mmol/L), respectively, and DF corresponds to the specific volumetric dilution factor executed during sample preparation.

### 2.6 Oil recovery

The fish oil obtained was characterized in terms of the acidity index (AI) (AOCS, Ca 5a-40) (25), which was measured according to the American Oil Chemists' Society. The peroxide value (PV) (AOCS, Cd 8–53) (25) and p-anisidine value (AV) (AOCS, Cd 18-90) (25) were carried out to evaluate the oxidation, and total oxidation was determined by the TOTOX index (TOTOX = 2PV + AV). Relative density was determined using a pycnometer calibrated at 20 °C. Color measurement of the oil samples was carried out using a PCE-CSM 7 Colorimeter. The instrument was standardized prior to each analytical execution utilizing official black and white reference calibration tiles to secure optical baseline accuracy. The results were expressed through L^*^, a^*^, and b^*^, where the L^*^ value is the “lightness” of a sample from 0 to 100, with 100 being pure white, the a^*^ value describes red (+) to green (–), and the b^*^ value represents yellow (+) to blue (–).

The fish oils were analyzed by gas chromatography to determine their fatty acid profile (AOAC 996.06) (14). The fatty acid methyl esters (FAMEs) were prepared and analyzed using a Thermo Scientific Trace ULTRA Series gas chromatograph with an autosampler and a flame ionization detector (GC-FID). The separation process was carried out using a Phenomenex ZB 5HT Inferno (30 m x 0.32 mm i.d.) coated with a 0.10 μm thickness film of 5% diphenyl-95% dimethylpolysiloxane. Injections were made in split-splitless mode (SSL) using hydrogen as the carrier gas (1 mL/min). All data were processed using Chrom-Card software. The retention times of the fatty acids were compared with those of chromatographic standards (Sigma-Aldrich: St. Louis, MO, United States), establishing stable integration conditions to resolve the relative percentage area of all major saturated, monounsaturated, and polyunsaturated omega-3 fatty acid tracks.

### 2.7 Formulation of the hybrid spread

Four innovative hybrid plant–marine spread formulations, along with one protein-free control sample were developed. To overcome the physical variations and structural limitations caused by freezing and high-pressure pasteurization side-effects, the thawed wet oat okara underwent a mechanical refinement process. No external water or thermal cooking was applied during this phase to preserve the original techno-functional properties of the structural residues. The wet biomass was treated using a high-shear laboratory homogenizer at 5,000 rpm for 3 min at room temperature (22 ± 1 °C). This mechanical conditioning was executed to break down large fiber aggregates and reduce particle size heterogeneity, resulting in a smooth, viscous, and highly cohesive structural paste suitable for multi-phase emulsion design.

The SPH was introduced in its liquid form, and its volumetric contribution to the continuous aqueous phase was strictly considered when adjusting the total water content of the formulations to maintain consistent moisture levels. The emulsification was conducted via a structured multi-step sequence. The designated liquid hydrolysate (SPH), xanthan gum, ascorbic acid, salt, and spices were completely dissolved and dispersed into the continuous aqueous phase (added water). This functional liquid phase was combined with the pre-conditioned wet oat okara structural paste inside a specialized reaction vessel.

The lipid phase (corn oil or corn/salmon oil blend) was subsequently added in a slow, continuous stream under continuous high-shear mechanical homogenization using an Ultra-Turrax rotor-stator system. The mixing speed was progressively accelerated to promote fine droplet breakdown and the structural formation of a stable oil-in-water O/W hybrid emulsion matrix with a smooth and creamy texture.

The complete matrix compositions (expressed in grams per 100 g of final product) are detailed in Table 1.

**Table 1:** Ingredient composition and experimental design of the developed plant–marine hybrid functional spreads.

Ingredient (g/100 g)	Control	System A SPH	System B SPH	System A SPH/SO	System B SPH/SO
Wet oat okara	38.00	38.00	38.00	38.00	38.00
Corn oil	19.00	19.00	19.00	16.00	16.00
Salmon oil SO	0.00	0.00	0.00	3.00	3.00
Liquid hydrolysate SPH	0.00	5.00	5.00	5.00	5.00
Added water	41.00	36.00	36.00	36.00	36.00
Xanthan gum	0.15	0.15	0.15	0.15	0.15
Ascorbic acid	0.20	0.20	0.20	0.20	0.20
Salt and spices	1.65	1.65	1.65	1.65	1.65
**Total**	**100.00**	**100.00**	**100.00**	**100.00**	**100.00**

Bold values indicate the total formulation percentage (100%).

### 2.8 Physicochemical analysis texture profile analysis (TPA) of the final spread

The finalized hybrid spreads were characterized according to their essential physicochemical baselines.

The chemical proximate composition was evaluated according the AOAC Official Method (2019). Total moisture content was calculated via gravimetric water loss at 105 °C, the crude protein was quantified via nitrogen analysis and total fat was determined via solvent extraction The pH profiles of the emulsified matrices were monitored using a digital laboratory pH meter (Crison Instruments S.A., Barcelona, Spain), which was inserted directly into the semi-solid matrix at 25 °C in accordance with the AOAC Official Method 981.12 (14).

The spreadable product was analyzed for texture using a TA.XT2 Texture Analyzer (Stable Micro Systems, Surrey, UK), which was equipped with a cylindrical P/35 probe. The sample was placed in standard cylindrical containers to ensure complete filling, while avoiding the presence of internal air pockets or headspace in order to minimize measurement artifacts. Texture profile analysis (TPA) was carried out using a double-compression test under controlled deformation conditions. Force–time curves were recorded and used to obtain the main textural parameters of the spreadable matrix, including force, area, time, hardness, adhesiveness, springiness, cohesiveness, gumminess, chewiness, and resilience following the standardized mechanical definitions established by Bourne (26) and the guidelines outlined in ISO 11036. Finally, the native color attributes of the finished spreads were recorded using a Minolta CR-400 colorimeter (Konica Minolta, Tokyo, Japan), which was calibrated under a standard D65 illuminant, under the international CIE L^*^a^*^b^*^ (27).

### 2.9 Statistical analysis

All experimental formulations and analytical characterizations were performed at least in duplicate. To secure strict statistical quality control, the Coefficient of Variance (CV) was dynamically monitored across runs; in cases where the CV exceeded the 5% threshold boundary, a strict triplicate was immediately executed and assayed. Experimental data were structurally reported as mean values ± standard deviation. The specific number of independent replicates assigned to each targeted analytical parameter is explicitly detailed within the captions and footnotes of the corresponding tables and figures. Statistically significant differences between the products obtained by enzymatic systems, and between the engineered hybrid functional spreads and the un-enriched vegetable control sample were systematically evaluated via one-way Analysis of Variance (ANOVA). Pairwise multiple comparisons were resolved utilizing appropriate *post-hoc* criteria, specifically Duncan's Multiple Range test, with the statistical significance threshold locked at *p* < 0.05. Statistical computations, variance models, and *post-hoc* alignments were managed using InfoStat processing software.

## 3 Results and discussion

### 3.1 Elemental and nutritional profiling of raw salmon trimmings

As shown in Table 2, a comprehensive characterization of raw Atlantic salmon (*Salmo salar*) trimmings established a baseline of highly concentrated macronutrients, with crude lipids accounting for 32.61% ± 1.80% of the dry weight, crude protein accounting for 63.08% ± 1.38%, and the mineral ash fraction resolving at 5.22% ± 0.46%. The proximate values obtained for the raw salmon material fall strictly within the characteristic concentration ranges previously compiled and reported by Ramakrishnan et al. (4) for industrial Atlantic salmon secondary streams, including both structural frames and meat-rich trimmings. This statistical alignment confirms the chemical authenticity and nutritional density of the processing by-products utilized in this bioreactor, reinforcing their suitability as robust macro-ingredient suppliers for circular food design.

**Table 2:** Proximate and elemental composition (dry basis) of raw Atlantic salmon (*Salmo salar*) trimming by-products.

Parameter	Content (% d.b.)^*a*^
Protein	63.08 ± 3.98
Lipids	32.61 ± 1.80
Ash	5.22 ± 0.46
N	10.09 ± 0.63
C	61.50 ± 0.82
H	9.74 ± 0.11
S	ND^*b*^

Values are expressed by mean value (n = 2) ± sd.

^*a*^d.b., dry basis.

^*b*^ND, non-detectable.

This slight mass-balance overlap is chemically and robustly explained by the elemental interactions captured via the CHNS combustion method. The raw matrix presented a dense carbon (C) fraction of 61.50% ± 0.82% and a hydrogen (H) fraction of 9.74% ± 0.11%, which structurally confirms the high prevalence of long-chain polyunsaturated fatty acids within the lipid core. Notably, total elemental nitrogen (N) was resolved at 10.09% ± 0.63%. Biochemically, a small amount of volatile elemental carbon and oxygen remains chemically complexed and fixed as inorganic salts (such as metal carbonates) during gravimetric calcination in the muffle furnace. This creates a small analytical overlap between the total carbon profile and the final mineral ash weight. Sulfur (S) remained below the limit of detection (nd), which is consistent with the low baseline of free sulfur-containing amino acids in the myofibrillar trimming stream. This nutrient-dense baseline highlights the potential of these salmon processing by-products to act as powerful texturizing and nutritional fortifiers in the hybrid spread matrix.

The absolute amino acid profile of the salmon trimming by-products revealed a high abundance of essential amino acids (EAA), particularly leucine, isoleucine, lysine, methionine, and phenylalanine, confirming the nutritional potential of these marine side-streams as high-quality protein ingredients (Table 3). His prominent EAA density aligns strictly with the nutritional boundaries compiled by Ramakrishnan et al. (4) for industrial Atlantic salmon processing side-streams. Furthermore, as highlighted by Aspevik et al. (28), the high concentration of branched-chain amino acids (BCAAs), led by leucine and isoleucine, underscores the biological value of this muscle-rich trimming stream, making it an exceptional ingredient. The considerable levels of glycine and hydroxyproline quantified in the chromatograms successfully validate the structural presence of collagen-rich connective tissues native to the residual skin and intramuscular sheets of the fileting streams, which may strategically influence the visco-elastic and functional properties of the resulting hydrolysates. Stoichiometrically, evaluating the true amino acid mass balance against the total nitrogen factor accumulation allowed for the calculation of an amino acid-derived nitrogen-to-protein conversion factor kp of 6.48 for these specific salmon trimming by-products. This derived value indicates a slightly higher true protein content within the muscular fragments than that estimated using the conventional default factor of 6.25. Nevertheless, the standard 6.25 baseline was retained for proximal analysis to facilitate direct mathematical comparison with previous literature and international nutritional studies.

**Table 3:** Detailed amino acid profile of raw Atlantic salmon (*Salmo salar*) trimming by-products.

Amino acid classification	Amino acid	Content (g/100 g salmon dry biomass)^*a*^
Essential (EAA)	Leucine (LEU)	8.73 ± 0.91
	Phenylalanine (PHE)	9.26 ± 0.75
	Lysine (LYS)	6.51 ± 0.60
	Isoleucine (ILE)	6.92 ± 0.72
	Valine (VAL)	3.75 ± 0.44
	Histidine (HYS)	2.68 ± 0.23
	Threonine (TRE)	3.86 ± 0.45
	Tryptophan (TRP)	3.07 ± 0.20
	Methionine (MET)	8.48 ± 0.77
Non-essential (NEAA)	Aspartic acid (ASP)	13.33 ± 1.37
	Glycine (GLY)	8.74 ± 1.68
	Alanine (ALA)	6.38 ± 1.01
	Serine (SER)	3.59 ± 0.48
	Proline (PRO)	3.16 ± 0.38
	Hydroxyproline (HYP)	3.21 ± 0.33
	Glutamic acid (GLU)	1.32 ± 0.12
	Glutamine (GLN)	1.09 ± 0.10
	Tyrosine (TYR)	3.95 ± 0.29
	Cysteine (CYS)	0.41 ± 0.05
	Hydroxylysine (HYL)	0.08 ± 0.01
	Arginine (ARG)	1.50 ± 0.12

Values are expressed by mean value (n = 2) ± sd.

^*a*^Data normalized as grams per 100 g of total recovered amino acid residues (derived from the corrected sum pool of 606.61 mg/g dry biomass).

### 3.2 Techno-functional performance of salmon protein hydrolysates (SPH)

The biochemical characterization of the liquid salmon protein hydrolysates confirmed significant differences (*p* < 0.05) between the two multi-enzyme systems evaluated as can be seen in Tables 4, 5.

**Table 4:** Recovery yields of hydrolysis products obtained from Atlantic salmon trimmings after enzymatic hydrolysis and centrifugation.

Parameter	System A (AL+ FL)	System B (PP + FL)
Hydrolysate recovered (mL)	838.30a ± 7.25	849.60a ± 2.25
Oil recovered (mL)	70.60a ± 2.0	72.20a ± 1.50
Protein recovered %	34.70b ± 0.28	27.0a ± 1.12

Values are expressed by mean value (n = 2) ± sd. The same letter in the row means the values of each beverage are not different by the Duncan test (*p* < 0.05).

a it means the minor value.

b it means the major value.

**Table 5:** Physicochemical and antioxidant properties of liquid salmon protein hydrolysates (SPH) obtained via sequential multi-enzyme systems.

Parameter	System A (AL+ FL)	System B (PP + FL)
Soluble protein (mg/mL)	27.87b ± 2.35	19.40a ± 1.27
Antioxidant capacity (mmol FeSO4/L)	2.11b ± 0.51	1.26a ± 0.20
Liquid density (g/cm^3^)	1.0225a ± 0.0033	1.0206a ± 0.0105
Solubilized solids (%)	2.25a ± 0.33	2.06a ± 1.05
TN (mg/L)	10450b ± 134	8020a ± 110
TC (mg/L)	27,190b ± 145	22,710a ± 130

Values are expressed by mean value (*n* = 2) ± sd. The same letter in the row means values of each beverage are not different by the Duncan test (*p* < 0.05).

a it means the minor value.

b it means the major value.

#### 3.2.1 Biocatalytic efficiency and solubilization behavior

Mathematical monitoring of nitrogen solubilization (DH_N_) revealed significant biocatalytic differences (*p* < 0.05) between the two multienzyme configurations. System A (AL + FL), which operates using the broad-specificity endopeptidase Alcalase^®^ 2.4 L, achieved a superior nitrogen cleavage efficiency, reaching an official DH_N_ threshold of 34.72% ± 1.28%, which is consistent with the high-value protein recovery yield of 32.25% documented in Table 4. In contrast, System B (PP + FL), with its more conservative exopeptidase cutting sequence, generated significantly lower nitrogen release, stabilizing at a DH_N_ baseline of 26.99% ± 1.12%.

This macro-solubilization behavior is cross-linked and strongly validated by independent spectrophotometric Lowry (37) profiles (Table 5). System A produced significantly higher soluble protein content (27.87 ± 2.35 mg/mL) than the conservative output of System B (19.40 ± 1.27 mg/mL). This operational consistency proves that extensive internal peptide bond cleavage, driven by Alcalase^®^ 2.4 L, successfully uncoiled the complex myofibrillar protein cages of the salmon trimmings and transferred highly soluble organic nitrogen to the aqueous phase (21). Importantly, this increased peptide solubility is directly correlated with the superior ferric reducing antioxidant power (FRAP = 2.11 ± 0.51 mmol FeSO4/L) observed in System A.

The results show that controlling the macro-solubilization window between 27% and 34% optimizes the presence of short-to-medium chain amphiphilic peptide surfactants. This provides the ideal molecular weight ceiling required for subsequent migration and structural reinforcement of continuous hybrid spread matrices.

It should be noted that protein concentrations estimated from total nitrogen measurements were higher than those determined by the Lowry (37) assay, which is expected due to the different analytical principles of both methods. Nevertheless, both analytical approaches followed the same trend, confirming the superior proteolytic performance of the AL + FL enzymatic system (System A) compared to the alternative PP + FL formulation (System B). These significant differences in soluble protein concentration are directly linked to the distinctive cutting behaviors established during processing. The broad-specificity interior cleavage characteristic of System A promoted a deeper structural breakdown of the salmon muscle, maximizing nitrogen solubilization. This prominent proteolytic efficiency is strongly aligned with the subsequent antioxidant boundaries, where the higher concentration of low-molecular-weight peptides directly enhanced the functional performance of the resulting marine extracts. This increases protein solubility and digestibility (16).

In contrast, Protana^®^ Prime is a proprietary blend of exopeptidases designed to release free amino acids from peptide ends. It combines aminopeptidase and carboxypeptidase activities. This improves palatability and nutritional value by enriching feed with amino acids (16, 17). Additionally, Flavourzyme^®^ 500 MG, contains both endo- and exopeptidase activities. Notably, it contains aminopeptidases and carboxypeptidases, enabling internal cleavage and the removal of terminal residues. This enhances flavor and reduces bitterness (16, 18).

#### 3.2.2 Antioxidant capacity

Significant differences in soluble protein concentration are directly linked to the antioxidant profiles determined using the FRAP assay. SPH obtained through System A (AL + FL) displayed a ferric reducing power of 2.11 ± 0.51 mmol FeSO4/L, almost doubling the performance of System B (PP + FL), 1.26 ± 0.20 mmol FeSO4/L. Sapatinha et al. (17) established that cascading enzymatic systems applied to fish-processing co-products significantly elevate the presence of low-molecular-weight bioactive peptides with strong radical-scavenging and reducing capabilities.

This suggests that extensive internal peptide cleavage by Alcalase^®^ 2.4 L successfully uncoiled the dense protein chains, exposing electron-donating hydrophobic residues and generating highly active low-molecular-weight bioactive peptides (16, 17). Within the hybrid food matrix, this enhanced reducing capacity represents a key technological asset to prevent lipid peroxidation and delay the oxidation of vulnerable omega-3 fatty acids.

#### 3.2.3 Free amino acid (FAA) profiles

**Figure 1:**
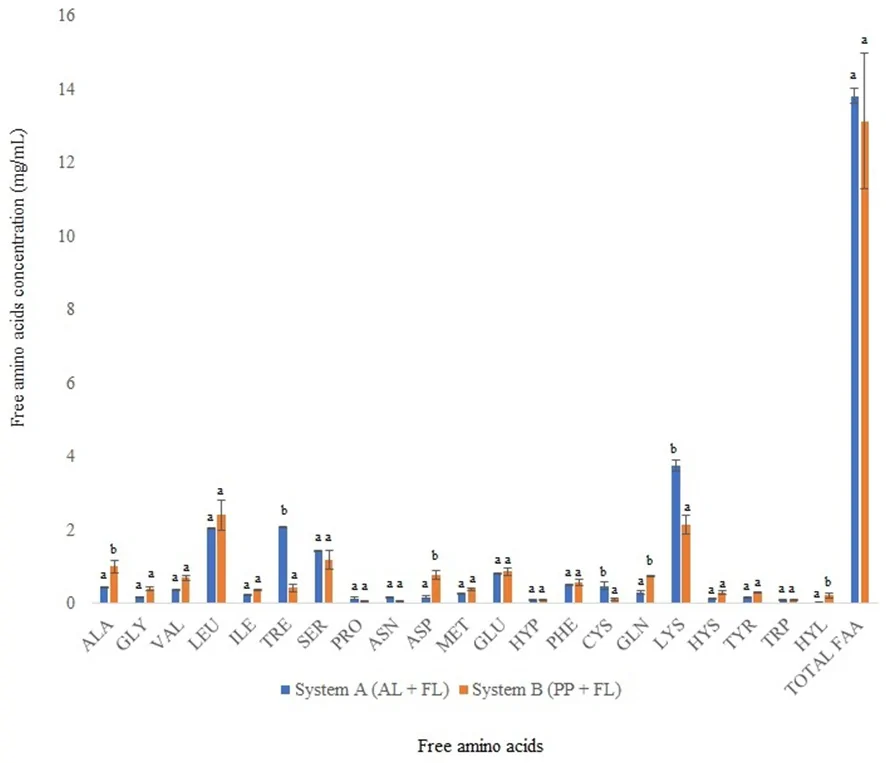
Free amino acid (FAA) composition of salmon protein hydrolysates produced by sequential hydrolysis with System A (Alcalase^®^ 2.4 L + Flavourzyme^®^) and System B (Protana^®^ Prime + Flavourzyme^®^). Values are expressed as mean ± standard deviation (*n* = 2). Different letters indicate significant differences between treatments for each amino acid (*p* < 0.05). Total FAA concentrations were 13.81 ± 0.20 mg/mL and 13.13 ± 1.85 mg/mL for Systems A and B, respectively.

The free amino acid (FAA) profiles of the liquid salmon protein hydrolysates were determined to evaluate the proteolytic performance of the two sequential enzymatic systems (Figure 1). Both treatments generated substantial concentrations of soluble FAAs, confirming extensive protein hydrolysis of the salmon trimming substrates. System A (AL + FL) produced a slightly higher cumulative FAA concentration (13.81 ± 0.20 mg/mL) than System B (PP + FL) (13.13 ± 1.85 mg/mL).

While minor fluctuations in individual FAA concentrations are inherently expected when processing complex and heterogeneous marine co-products owing to seasonal and batch-to-batch variations in the raw salmon trimmings (28), the overall distribution patterns remained highly consistent between both treatments. This behavior is primarily explained by the shared second hydrolysis stage using Flavourzyme^®^ 500 MG. This commercial enzyme complex contains both endo- and exopeptidase (aminopeptidase and carboxypeptidase) activities, enabling progressive hydrolysis of the intermediate peptides generated in the first stage (18). Consequently, Flavourzyme^®^ acted as a biochemical equalizer, reducing major discrepancies in total FAA accumulation while preserving subtle, distinct amino acid fingerprints generated by the primary proteases (Alcalase^®^ 2.4 L or Protana^®^ Prime) (17).

A comprehensive characterization of the total amino acid profile was not deemed essential, as enzymatic hydrolysis does not modify the elemental amino acid composition of the raw substrate but rather alters the molecular weight distribution and the ratio between protein-bound and free amino acid fractions (28). Interestingly, despite the similar total FAA levels, System A exhibited significantly higher antioxidant activity. This confirms that the antioxidant capacity of these hydrolysates cannot be attributed solely to free amino acid concentrations. Instead, it is highly dependent on peptide sequence composition, molecular weight distribution, and the synergistic presence of specific bioactive peptides and terminal amino groups capable of participating in single-electron transfer reactions (29). Overall, these results demonstrate that controlling the macro-solubilization window (between 27% and 34%) and selecting the appropriate primary endopeptidase optimizes both the bioactivity and the surfactant properties of the resulting ingredients, making them highly suitable for the stabilization of continuous hybrid food matrices.

### 3.3 Methodological evaluation of the salmon lipid fraction

The oil obtained from salmon trimmings by Bligh and Dyer (32.61%) is consistent with the data reported by Haq et al. (39) for trimmed muscle on a dry matter basis. Using this percentage as the total yield, the enzyme systems AL + FL and PP + FL produced yields of 86.90% and 88.86%, respectively. These results are slightly higher than those reported by Deepika et al. (30) for salmon frames treated with enzymes, and consistent with the results reported by Haq et al. (39) for CO_2_ extraction.

The physicochemical parameters are summarized in Table 6. The acidity of the oil is an important quality parameter related to the presence of free fatty acids (FFA) and other non-lipid compounds. The presence of FFAs is mainly due to triacylglycerol hydrolysis reactions. Non-lipid material, such as acetic acid, is produced during the degradation of the raw material. Thus, acidity depends on factors related to the oil's composition, such as the extraction procedure and the freshness of the raw material (31). In the present study, the acidity value of the oil was lower when both enzymes were used. Similar results were reported by Deepika et al. (30) for Salmo salar frames using the same extraction methods.

**Table 6:** Physicochemical quality attributes, hydrolytic acidity, and oxidative stability indicators of salmon oils recovered via cascading enzymatic treatments.

Parameter	System A (AL + FL)	System B (PP + FL)
Acidity % oleic acid	0.47b ± 0.03	0.40a ± 0.02
Peroxide value (meq/kg)	1.41a ± 0.01	1.44b ± 0.03
p-anisidine	1.80a ± 0.09	1.63a ± 0.09
TOTOX	4.62a ± 0.11	4.51a ± 0.15
Density (g/cm^3^)	0.954a ± 0.023	0.961a ± 0.010
Color		
L^*^	38.69a ± 0.21	38.74a ± 0.33
a^*^	5.66a ± 0.22	6.88b ± 0.31
b^*^	2.42a ± 0.03	2.45a ± 0.03

Values are expressed by mean value (*n* = 2) ± sd. The same letter in the row means values of each beverage are not different by the Duncan test (*p* < 0.05).

a it means the minor value.

b it means the major value.

The peroxide value (PV) is a conventional method of quantifying the total hydroperoxides produced by primary oxidation during thermo-oxidative or lipid oxidation processes (32). These oxidation products then decompose to form lower-molecular-weight compounds, such as free fatty acids, alcohols, and aldehydes (33). The PV is used to determine the extent of secondary oxidation in the oil and quantify aldehydes with α- and β-unsaturation. The values obtained in this study were less than 2 meq/kg in both extraction systems, consistent with values reported by Haq et al. (39) and Deepika et al. (30) for salmon trimmings and frames. The Global Organization for EPA and DHA (GOED) (35) and the United Nations Food and Agriculture Organization (FAO) (36) have set a maximum permissible PV limit of 20 meq/kg for fish oils to be considered acceptable and fit for human consumption. The results of the present study indicated that the anisidine values of all oil samples were below the permitted limit of 20. These values are slightly lower than those reported by Haq et al. (39) and much higher than those reported by Deepika et al. (30) for salmon frame oil obtained using enzymes. Therefore, Boran et al. (33) studied the effect of storage temperature and time on the quality of several fish oils and found that the anisidine value ranged from 1.74 to 14.09. Thus, the value obtained indicates the freshness and correct handling of the raw material.

**Figure 2:**
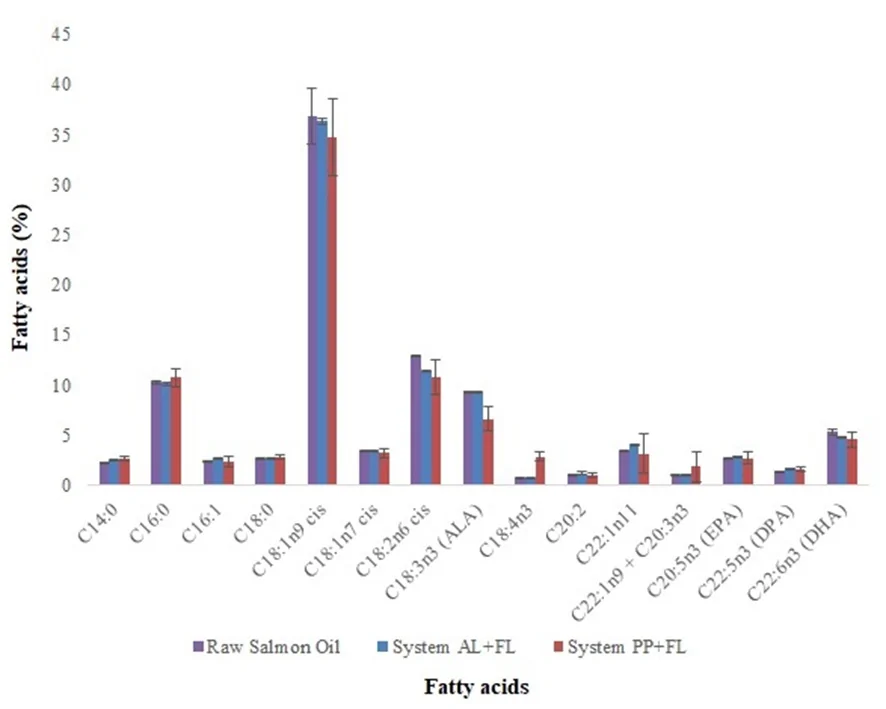
Fatty acid methyl ester (FAME) composition of the lipid fraction from raw Atlantic salmon (*Salmo salar*) trimmings and oils recovered after sequential enzymatic hydrolysis using Alcalase^®^ 2.4 L + Flavourzyme^®^ (AL+FL) and Protana^®^ Prime + Flavourzyme^®^ (PP+FL). Values are expressed as mean ± standard deviation (*n* = 4), with error bars on the graph representing the corresponding standard deviations across replicates. Data are expressed as the relative percentage of the total integrated peak area (%) of total identified FAMEs. Minor unidentified fatty acid tracks (< 0.5%) representing less prevalent isomers or trace fractions were excluded from the final data consolidation to maximize analytical clarity.

Comparative screening of the fatty acid methyl ester (FAME) profiles across all extraction trials demonstrated outstanding operational stability. The SPH oils recovered through Systems A (AL + FL) and B (PP + FL) strictly mirrored the core macromolecular signature of the raw Atlantic salmon trimmings, with exceptionally low standard deviations across replicates (Figure 2). Oleic acid (C18:1n9 cis) consistently emerged as the primary monounsaturated lipid anchor, achieving concentrations of 34.00 ± 0.00% and 34.55 ± 0.05% in Systems A and B, respectively, and maintaining a close balance with the native baseline of 35.54% ± 0.31%. Saturated palmitic acid (C16:0) was found to be precisely at 9.49% (System A) and 9.67% (System B).

Crucially, there were no statistical differences or structural degradation in the highly sensitive long-chain marine omega-3 cluster when shifting from the raw tissue to the enzyme-liberated fractions. Alpha-linolenic acid (C18:3n3, ALA) stabilized at around 8.8%, while docosahexaenoic acid (C22:6n3, DHA) consolidated at 4.63% (System A) and 4.68% (System B), which matched the configuration of the raw matrix (5.03%). This unaltered and highly reproducible lipid architecture proves that both Alcalase^®^ 2.4 L and Protana^®^ Prime cascading operations function as secure, non-destructive bio-extraction mechanisms. The enzymatic actions successfully de-emulsify the structural protein cages to release the lipid core without inducing thermo-oxidative stress or lipid isomerization, providing a standardized, omega-3-enriched functional ingredient for hybrid circular food design.

### 3.4 Compositional and elemental characterization of okara raw materials

The elemental analysis of oat okara revealed a composition of 46.76% ± 0.41% carbon, 7.15% ± 0.08% hydrogen and 4.36% ± 0.21% nitrogen, while sulfur remained below the detection limit. Based on the experimentally determined nitrogen-to-protein conversion factor, the crude protein content was estimated at 25.74% ± 0.98% on a dry-weight basis. The material exhibited a dry matter content of 57.06% ± 0.02%, corresponding to a moisture content of 42.94% ± 0.02%. Residual lipids and ash accounted for 11.03% ± 0.08% and 5.96% ± 0.12%, respectively.

The detailed compositional characterization obtained following NREL analytical procedures is summarized in Table 7. Water- and ethanol-soluble extractives represented 15.07% ± 0.80% and 4.97% ± 0.50% of the biomass, respectively, indicating that a substantial fraction of the original oat constituents remained in soluble form after beverage production. The structural fraction was dominated by lignin, with acid-insoluble (Klason) lignin accounting for 15.13% ± 0.93% and acid-soluble lignin for 7.15% ± 0.28%, resulting in a total lignin content of 22.28%.

**Table 7:** Comprehensive compositional characterization of oat okara biomass, including extractives, structural carbohydrates, lignin, protein, lipids, and ash contents (dry weight basis).

Compound	Composition, % (dry base)
Water extractives	15.07 ± 0.8
Ethanol extractives	4.97 ± 0.5
Acid-soluble lignin	7.15 ± 0.28
Acid-insoluble lignin	15.13 ± 0.93
Cellulose (Glucans)	5.6 ± 0.4
Hemicellulose (Xyl+Arab+Acet)	Xilanos 4.06 ± 0.59
	Arabinanos 2.53 ± 0.38
	Acetyl 0.34 ± 0.04
Crude protein	25.74 ± 0.98
Crude lipids	11.03 ± 0.8
Mineral ash	5.96 ± 0.12
Total closure	**97.58%**

Values are expressed by mean value (*n* = 2) ± sd. The bold value highlights the TOTAL CLOSURE (97.58%).

Structural carbohydrates represented 12.53% of the dry biomass and consisted mainly of glucans (5.60% ± 0.40%), xylans (4.06% ± 0.59%), arabinans (2.53% ± 0.38%), and acetyl groups (0.34% ± 0.04%). These results indicate that, despite the extraction processes involved in oat beverage manufacture, the remaining okara retained a significant fibrous network capable of contributing to water retention and structural stabilization in food systems.

Overall, the integration of all quantified fractions resulted in a mass-balance closure of 97.58%, demonstrating excellent analytical recovery and internal consistency of the compositional characterization. The small unrecovered fraction (2.42%) can be attributed to minor analytical uncertainties, non-protein nitrogen compounds, and structural transformations occurring during acid hydrolysis.

### 3.5 Physicochemical characteristics of hybrid spreads

The final hybrid spreads exhibited a relatively homogeneous physicochemical profile, with moisture values ranging from 16.12% to 18.52% and pH values close to neutrality (6.84–7.07) (Table 8). This indicates high compatibility between the oat okara matrix and the incorporated salmon protein hydrolysates (SPH). The slightly higher moisture content observed in SPH-enriched formulations may be due to the water-binding capacity of low-molecular-weight peptides interacting with the hydrophilic polysaccharide network of oat fibers.

**Table 8:** Comprehensive physicochemical, proximate, colourimetric, and instrumental textural profiles of the engineered plant–marine hybrid functional spreads.

Formulation	Moisture (%)^1^	Crude protein (% d.b.)^2^	Crude fat (% w.b.)^3^	pH	Hardness (N)4	L^*^	a^*^	b^*^
Control (Oat base)	56.12a ± 1.90	15.48a ± 2.33	7.43b ± 0.43	6.99a ± 0.02	3.83c ± 0.45	59.31ab ± 1.70	4.26b ± 2.17	17.26ab ± 1.60
System A SPH	58.08b ± 2.12	17.58bc ± 1.46	6.17a ± 0.50	6.88a ± 0.01	2.71b ± 0.74	62.99b ± 0.31	2.57a ± 0.05	16.97a ± 0.79
System B SPH	58.40b ± 0.91	17.28bc ± 2.20	6.24a ± 0.19	6.84a ± 0.02	1.79a ± 0.58	62.26b ± 0.14	2.66a ± 0.08	16.81ª ± 0.20
System A SPH/SO	58.52a ± 0.23	24.07d ± 2.34	6.04a ± 0.04	7.07a ± 0.02	3.11bc ± 0.58	62.19b ± 0.45	2.88a ± 0.17	16.95ª ± 0.38
System B SPH/SO	57.94b ± 1.73	20.45c ± 2.56	6.37a ± 0.46	7.01a ± 0.01	3.94c ± 0.96	64.05bc ± 0.56	3.04a ± 0.03	18.29b ± 0.11

Values are expressed by mean value (*n* = 2) ± sd. Different letters within the same column indicate statistically significant differences according to Duncan's test (*p* < 0.05), where “a” represents the lowest value and “d” represents the highest value.

^1^Residual moisture content within the stabilized dry-matter extracts before elemental and nitrogen profiling.

^2^d.b.: dry basis; derived utilizing the validated specific weighted nitrogen-to-protein conversion factor.

^3^w.b.: wet basis; represents the net lipid concentration within the finalized semi-solid spread matrices.

^4^Maximum force registered during the initial instrumental compression cycle.

Protein enrichment was evident after the incorporation of SPH. The control oat-based formulation had a protein content of 15.48% (d.b.), whereas adding hydrolysates increased this value to 24.07% in the System A SPH/SO formulation. This demonstrates the effectiveness of marine peptides as nutritional fortifiers in hybrid plant–marine matrices.

The crude fat content remained relatively stable across the formulations (6.04%−7.43% w/b), suggesting that partially replacing sunflower oil with recovered salmon oil did not significantly alter the spreads' overall lipid content. This suggests that the lipid phase could be reformulated to include recovered marine oil while maintaining a comparable total fat level.

#### 3.5.1 Texture and color properties

The incorporation of liquid SPH significantly reduced the instrumental hardness of the control vegetable formulation from 3.83 N down to 2.71 N and 1.79 N for Systems A and B, respectively (Table 8). This reduction indicates a clear plasticizing effect exerted by the small marine peptides within the oat fiber network. These low-molecular-weight fractions efficiently interfere with the intermolecular hydrogen bonding and steric interactions between raw oat polysaccharide chains (cellulose and arabinoxylans), thereby reducing the initial stiffness of the continuous phase and generating a softer, highly spreadable semi-solid cream.

Interestingly, the strategic reintroduction of 3% upcycled salmon oil partially restored the mechanical strength of the multi-phase systems, increasing hardness to 3.11 N in System A SPH/SO and reaching 3.94 N in System B SPH/SO. This rheological recovery confirms that the highly dispersed lipid droplets operate as active fillers embedded within the continuous protein–polysaccharide matrix reinforcing the structure through physical droplet-matrix interfacial anchorage (34). Supported by the surfactant action of the amphiphilic SPH strands, these microdroplets adhere to the micro-milled oat cell walls, reinforcing the biopolymer network through interfacial interactions. It is worth noting that while the maximum hardness of System B SPH/SO (3.94 N) numerically matches and slightly exceeds the baseline vegetable control (3.83 N), this structural shift does not imply a return to the initial rigid, brittle, and rubbery polysaccharide conformation. Instead, the oil-filled matrix transitions into a highly cohesive, viscoelastic emulsion network. This macrostructural arrangement translates into enhanced structural plasticity, smoothness, and desirable spreadable creaminess rather than an undesirable elastic stiffness, successfully satisfying the targeted design constraints.

Regarding the physical stability of these hybrid architectures, the formulations exhibited robust macrostructural integrity, with no observable phase separation, oil bleeding, or serum syneresis during processing or subsequent storage. This macroscopic stability is directly attributed to the efficient structural integration between the upcycled salmon oil and the continuous phase. The structural network, reinforced by the thickening capacity of xanthan gum and the micro-milled oat fiber matrix, effectively entraps the dispersed lipid droplets. Concurrently, the amphiphilic short-chain peptides within the liquid SPH act as interfacial surfactants, stabilizing the oil-water boundaries. This dual mechanism ensures that the lipid and aqueous fractions remain microstructurally bound within the complex biopolymer network, preventing physical destabilization without the need for synthetic emulsifiers.

Color analysis revealed a significant increase in lightness (L^*^) after SPH incorporation, peaking in the formulation containing System B SPH and salmon oil specifically (L^*^ = 64.05). This increase in lightness may be related to changes in the emulsion's microstructure, since the interactions between dispersed lipid droplets and the protein–polysaccharide matrix can enhance light scattering and surface reflectance. Therefore, incorporating SPH and recovered salmon oil not only modifies the mechanical properties of the spreadable matrix, but also contributes to a brighter visual appearance. Similarly, Pinar-Escobar et al. (12) reported that incorporating structured marine side-streams into fish-based pâtés enhanced structural stability and modulated viscosity, directly influencing product firmness and cohesion. These findings support the potential of combining upcycled marine and plant co-products, such as salmon residues and oat okara, to develop sustainable, spreadable emulsions with tailored rheological and optical properties.

Despite these promising findings, some limitations should be noted. While texture, color, and instrumental physicochemical parameters provide useful information on the technological performance of the spreads, the products were not evaluated through sensory analysis or consumer acceptability studies. Furthermore, the products were assessed for structural performance immediately after production; therefore, the long-term stability of the products during storage requires further investigation.

Future research should focus on advanced rheological characterization, including flow behavior, viscoelastic properties, and spreadability under different serving conditions. Accelerated and real-time storage studies will also be necessary to evaluate the physical stability, phase separation, and oxidative stability of the recovered marine lipid fraction. Finally, sensory evaluation and consumer acceptability studies will be essential to validate the organoleptic quality, flavor optimization, and practical applicability of these circular spreadable products.

## 4 Conclusions

This investigation successfully demonstrated the technical feasibility of co-valorizing industrial fish and agro-industrial side-streams, specifically Atlantic salmon fileting trimmings and pasteurized oat okara, into high-value; nutrient-dense hybrid functional spreads. Cascading enzymatic bioreactions successfully unlocked a double valorization vector, recovering around 30% of highly nutrient protein fractions while simultaneously isolating an ultra-fresh, un-oxidized marine lipid core rich in bioavailable omega-3 fatty acids (EPA and DHA).

Technologically, the amphiphilic salmon peptides operated in rheological harmony with the continuous structural oat cellulose and arabinoxylan fibers. While pure peptide fractions operated as structural plasticizers to disrupt polysaccharide self-aggregation and decrease continuous rigidity down to 1.79 N, the strategic reincorporation of 3% upcycled salmon oil operated as an active filler. Backed by the surfactant properties of the SPH, these micro-droplets anchored firmly onto the micro-milled plant cell walls, balancings instrumental hardness (1.79 N to 3.94 N), maximizing emulsified lightness (L^*^ = 64.05), and eliminating macrostructural phase separation, serum syneresis, or lipid bleeding.

## Data Availability

The raw data supporting the conclusions of this article will be made available by the authors, without undue reservation.
